# Comparative Safety of Anticoagulant, Antiplatelet and the Combination of Both for Acute Coronary Syndrome: A Systematic Review and Network Meta-Analysis

**DOI:** 10.3390/biomedicines13082027

**Published:** 2025-08-20

**Authors:** Qingsheng Niu, Ziyi Zhu, Fulin Wang, Yaowen Jiang

**Affiliations:** 1 Department of Emergency Medicine, Laboratory of Emergency Medicine, West China Hospital of Sichuan University, West China School of Medicine, Chengdu 610041, China; 2023324025243@stu.scu.edu.cn (Q.N.); 13165280302@163.com (F.W.); 2 Disaster Medical Center, Sichuan University, Chengdu 610041, China; 3 West China School of Medicine, Sichuan University, Chengdu 610041, China; zhuziyi@stu.scu.edu.cn

**Keywords:** acute coronary syndrome, anticoagulant, antiplatelet, safety, network meta-analysis

## Abstract

**Background:** Antithrombotic therapy plays an important role in acute coronary syndrome (ACS). The combination of anticoagulant and antiplatelet therapy resulted in fewer complications and stronger potency compared to traditional monotherapy. Our net meta-analysis aimed to compare and rank the safety of different treatments used in patients with ACS. **Method:** We conducted a search for trials in three prominent databases. The main objective of our investigation was to assess hemorrhage. Additional outcomes included mortality, myocardial infarction, stroke, and embolism. We used a frequentist network meta-analysis with a random-effects model to, directly and indirectly, compare safety across different antithrombotic strategies. **Result:** A total of 30 randomized clinical trials were included in this net meta-analysis with 135,471 ACS patients. In these eight different antithrombotic therapies, SAPT (single-agent platelet inhibitor therapy) showed the lowest risk of bleeding (SUCRA = 0.5%). The highest risk of bleeding was observed in VKA (vitamin K antagonists) + DAPT (dual antiplatelet therapy) (SUCRA = 99.8%). Bleeding among NOAC (non-vitamin K antagonist oral anticoagulants) + DAPT was found to be higher than DAPT (OR = 1.94, 95% CI = 1.42–2.65). NOAC + SAPT significantly reduced the embolism (OR = 1.50, 95% CI = 1.16–1.94) and myocardial infarction (OR = 1.22, 95% CI = 1.08–1.37) events compared with SAPT. In addition, VKA significantly reduced the rate of stroke compared with SAPT (OR = 3.45, 95% CI = 1.17–10.18). However, no significant difference was observed in death events among these eight antithrombotic therapies. **Conclusions:** We advise against the use of SAPT in ACS due to its elevated risk of embolism, myocardial infarction, and stroke. It is important to mention that the combination of NOAC and SAPT has a lower incidence of myocardial infarction, bleeding and embolism problems. Therefore, the combination of NOAC and SAPT may be the optimal approach to achieve a balance between the risks of bleeding and embolism. This meta-analysis was registered in PROSPERO with the registration number CRD42024542826.

## 1. Introduction

Acute coronary syndrome arises from the rupture or erosion of unstable atherosclerotic plaques within the coronary arteries, leading to thrombus formation and subsequently causing an acute myocardial ischemic syndrome. Of course, approximately 14% of overall patients with ACS failed to survive [1]. It is notable that the elderly have the highest incidence of cardiovascular disease and frequently present with ACS. However, recent studies showed that increasing incidence and presence were observed in younger individuals with ACS. This emerging phenomenon significantly contributes to the substantial economic burden that ACS imposes on society [2]. Long-term antiplatelet and anticoagulant therapies play a key role in preventing complications associated with ACS. Oral antiplatelet therapy (aspirin or P2Y12 inhibitors) and anticoagulants (unfractionated heparin, low-molecular-weight heparins, direct thrombin inhibitors, or Xa factor inhibitors) are recommended therapeutic approaches in the initial management of ACS, regardless of whether the treatment is invasive or non-invasive [3].

When ACS patients are exposed to antithrombotic therapy, bleeding is a known complication of any oral anticoagulant and antiplatelet medications [4]. Thrombotic risk is higher than bleeding risk in the early phase within the initial 30 days following ACS events. Next, risk factors for bleeding will transcend thrombus formation in the later stage with the implementation of intensified antithrombotic therapies, which results in attenuation of the benefits of anticoagulation. Hence, the chronic management of ACS requires a personalized approach to antithrombotic therapy following the acute phase to balance the risk of bleeding and embolism [5]. Although various studies revealed that novel oral anticoagulants overcome the drawbacks associated with traditional anticoagulants, including unfractionated and low-molecular-weight heparins, and vitamin K antagonists [6], anticoagulant or antithrombotic monotherapy cannot significantly benefit ACS patients compared to their combination [7]. Thus, increasingly, research has explored the safety and efficacy of novel oral anticoagulants in combination with standard antiplatelet therapy for ACS. These studies suggest that the combination of novel oral anticoagulants with antiplatelet therapy significantly reduces the occurrence of fatal bleeding and indicates the importance of incorporating low-dose novel oral anticoagulants in antiplatelet regimens [8]. This network meta-analysis systematically evaluated the safety of various combinations of novel or traditional anticoagulants with dual or single antiplatelet agents in ACS. The findings present novel insights into the treatment of ACS, offering new references for clinical practice.

## 2. Material and Method

This study conducted a systematic review and network meta-analysis in accordance with the guidelines provided by the Preferred Reporting Items for Systematic Reviews and Meta-Analyses extended statement for Network Meta-analyses (PRISMA-NMA) [9].

### 2.1. Search Strategy

This literature research was conducted for clinical trials from inception to 4 April 2025. Our research took place in three major databases, including PubMed/MEDLINE, Cochrane/CENTRAL, and Scopus, and was limited to papers published in the English language. The search strategy was as follows: (apixaban OR edoxaban OR darexaban OR rivaroxaban OR otamixaban OR direct oral anticoagulants) AND (myocardial ischemia OR acute coronary syndrome OR PCI OR coronary disease OR MI) AND (aspirin OR P2Y12 receptor antagonists OR antiplatelet).

### 2.2. Inclusion and Exclusion Criteria

Studies were included for the following reasons: (1) randomized controlled trials compared anticoagulant, antiplatelet or combination therapy, (intervention group or control group) in patients with coronary heart disease; (2) patients were administered VKA, NOAC, aspirin or P2Y12 receptor antagonists after coronary heart disease; (3) efficacy and safety endpoints.

Studies were excluded for the following reasons: (1) letters to the editor, reviews, and animal studies; (2) there was a combination of heparin or other non-antithrombotic interventions; (3) the studies were duplicates; (4) not RCT studies.

### 2.3. Outcome Assessment

Bleeding, death, myocardial infarction, stroke, and embolism were the main outcomes in this net meta-analysis. The safety of antithrombotic drugs was assessed by the rate of bleeding induced by antithrombotic drugs including major bleeding and clinically relevant non-major bleeding. Other outcomes (death, myocardial infarction, stroke, and embolism) represented the side effects of the drug.

### 2.4. Data Extraction and Assessment of Quality

The identified studies were imported into Endnote X9. Initially, we eliminated redundant studies. Two autonomous researchers (Qingsheng Niu and Ziyi Zhu) conducted data extraction and assessed the quality of references, followed by a cross-validation process. A third researcher (Fulin Wang) was included to conduct the discussion and reach a consensus if there was any disagreement. Basic information, inclusion and exclusion criteria, type of research, sample size, type and dosage of intervention treatment, control group, follow-up and outcome indicators were recorded. A method recommended in the Cochrane Handbook 5.1.0 was used to evaluate the quality [10]. The following aspects were examined: (i) allocation concealment, (ii) sequence generation, (iii) blinding of outcome assessment, (iv) blinding of participants and personnel, (v) selective reporting, (vi) incomplete outcome data, and (vii) other bias.

### 2.5. Statistical Analysis

Stata software version 17 (StataCorp LLC, College Station, TX, USA) was used for the data net meta-analysis (NMA). STATA packages used include mvmeta, network, st0411, and sencode package. Assessment of heterogeneity was done using RevMan 5.3. The rates of events with each antiplatelet, anticoagulant or combination treatment were entered as an individual study arm, and data were pooled in a multiple treatment NMA that allows integration of direct and indirect comparisons. Heterogeneity was also quantified using chi-square tests and the inconsistency statistic (I^2^). Heterogeneity was considered significant for values of *p* < 0.1 and I^2^ > 50%. When a moderate or high heterogeneity (I^2^ > 50% and *p*-value < 0.1) was observed, a random-effect model was employed; otherwise, a fixed-effect model was applied [11]. Odds ratio (OR) with confidence interval (CI) of 95% was adopted as a representative measure of dichotomous outcomes. The level of statistical significance was set as *p* < 0.05. The area under the cumulative ranking (SUCRA) showed the possibility of each intervention being the best.

## 3. Results

### 3.1. Search Results and Characteristics of Included Studies

**Figure 1:**
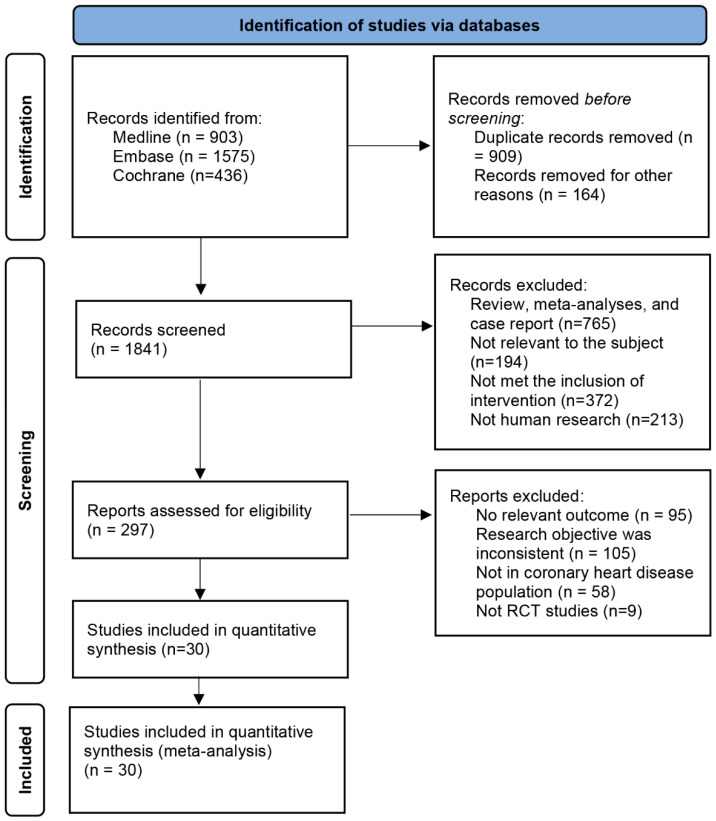
Process for identifying studies eligible for the network meta-analysis.

A total of 2914 articles were retrieved from Medline, Embase, and Cochrane databases. A number of 909 duplicate articles were eliminated using the EndNote and were excluded. On careful assessment of titles and abstracts, 1975 did not qualify according to the inclusion criteria and were excluded. Eventually, 30 RCTs studies were included in this net meta-analysis. Literature retrieval and screening processes were carried out in accordance with the PRISMA guidelines, the special process screening is shown in Figure 1, eight types of interventions were included: VKA, NOAC, DAPT, SAPT, VKA + DAPT, VKA + SAPT, NOAC + DAPT, NOAC + SAPT.

**Figure 2:**
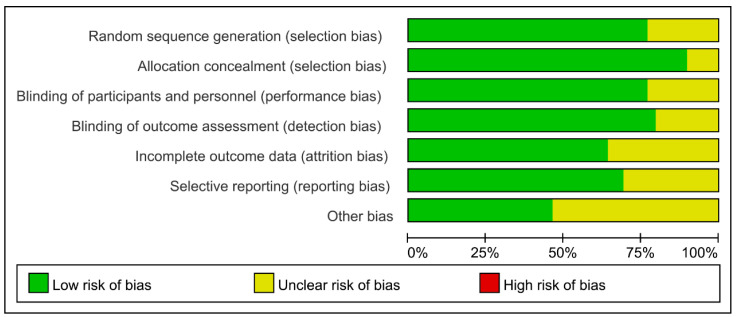
Methodological quality evaluation of the included studies.

Thirty studies with 135,471 patients with atrial fibrillation, ACS, or stable cardiovascular disease were included. The typical characteristics of the studies included are presented in Table 1. The methodological qualities of the included studies are presented in Figure 2. Table 2 illustrates the absolute event rates across various clinical outcomes, highlighting the differences between intervention and control groups.

### 3.2. Effect on Bleeding Event of Different Treatments

**Figure 3:**
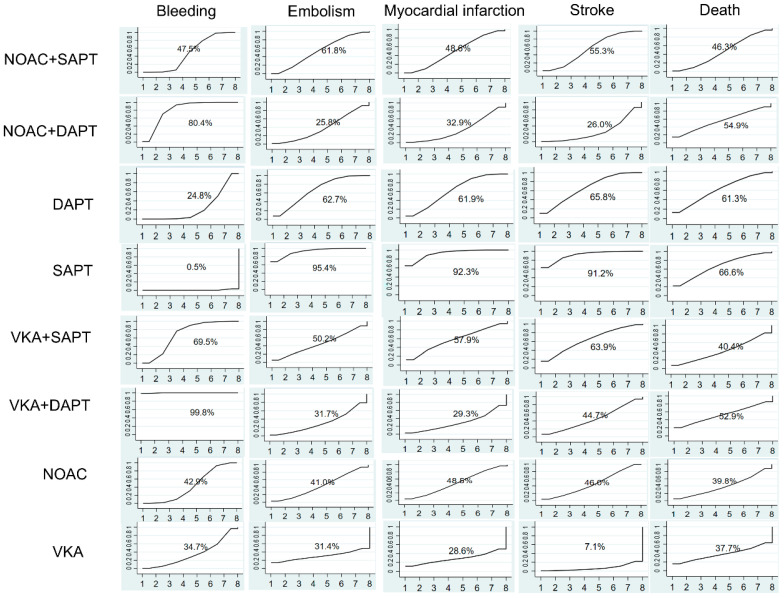
Ranking of treatment strategies based on probability of their protective effects on outcomes of bleeding, embolism, myocardial infarction, stroke, and death according to the cumulative ranking area (SUCRA).

**Figure 4:**
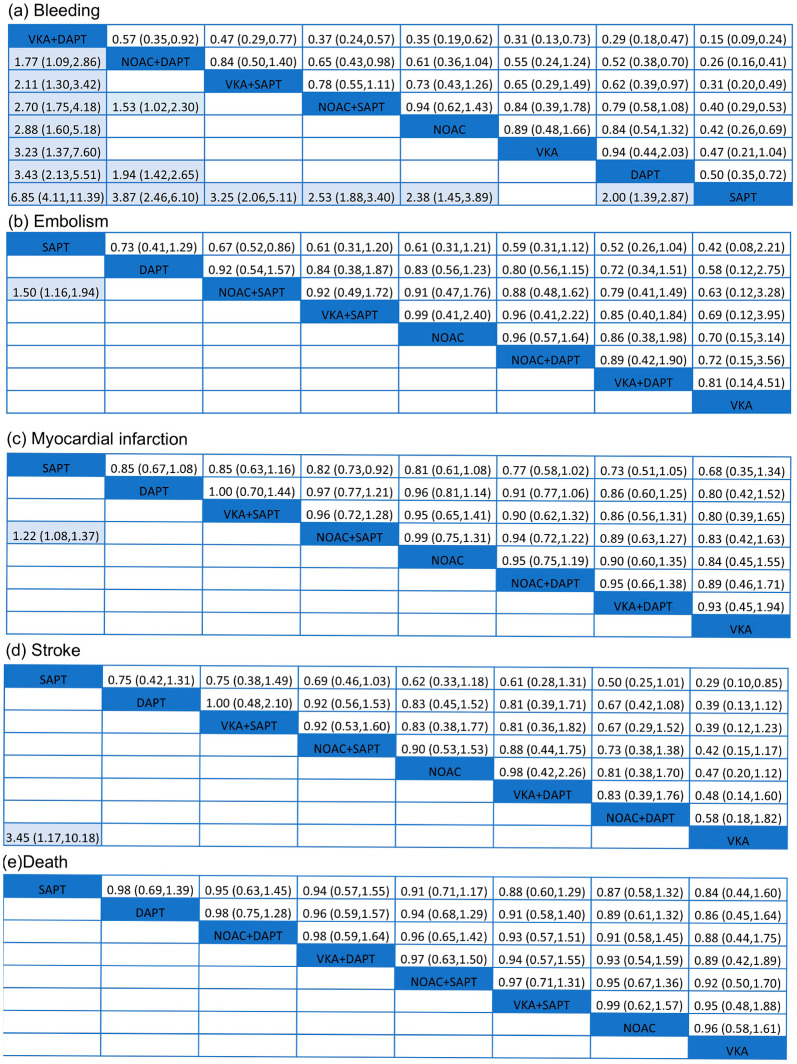
Summary of results from network meta-analysis (on the lower triangle) and traditional pairwise meta-analysis (on the upper triangle) on primary outcomes: (**a**) bleeding, (**b**) embolism, (**c**) myocardial infarction, (**d**) stroke, (**e**) death. On the lower triangle and upper triangle, the row-defining treatment is compared with the column-defining treatment, and ORs > 1 favor the row-defining treatment. Remarkable results are shown in shadow.

Data on the impact of combining anticoagulant medications with antiplatelet therapy on bleeding events were obtained from 29 trials [13,14,15,16,17,18,19,20,21,22,23,24,25,26,27,28,29,30,31,32,33,34,35,36,37,38,39,40,41] including a total of 97,478 participants. Figure 3 demonstrated a significantly elevated risk of bleeding in VKA + DAPT across all eight treatments, with a SUCRA value of 99.8%. Nevertheless, Figure 4a did not show any notable disparity between SAPT and VKA, with an odds ratio (OR) of 2.12 and a 95% confidence interval (CI) ranging from 0.96 to 4.69. The results indicated that the combination of standard anticoagulants with antiplatelet medications significantly increased the occurrence of bleeding complications. Furthermore, the incidence of bleeding in patients receiving NOAC + DAPT was observed to be greater compared to those receiving NOAC + SAPT, with an odds ratio of 1.53 and a 95% confidence interval ranging from 1.02 to 2.30. The lowest risk of bleeding was observed in SAPT (SUCRA = 0.5%).

### 3.3. Effect on Embolism Event of Different Treatments

Embolism events were reported from a total of 16 studies [17,18,19,21,22,23,25,26,27,29,30,31,33,36,37,41] of 75,261 patients. As shown in Figure 3, two effective treatments for reducing embolism were VKA (SUCRA 31.4%) and NOAC + DAPT (SUCRA 25.8%). SAPT (SUCRA 95.4%) increased embolism in patients with ACS. NOAC + SAPT significantly reduced embolism events compared with SAPT (OR = 1.50, 95% CI = 1.16–1.94), and other treatments were not superior to SAPT.

### 3.4. Effect on Myocardial Infarction Event of Different Treatments

A total of 24 studies [12,13,15,16,17,18,19,21,22,23,24,25,26,27,28,29,30,31,33,35,36,38,40,41] of 93,708 patients showed that SAPT experienced a high rate of myocardial infarction (SUCRA 95.6%). Figure 4c showed that, compared with SAPT, NOAC + SAPT (OR= 1.22, 95% CI = 1.08–1.37) decreased myocardial infarction in patients with ACS. For reduction on myocardial infarction, VKA (SUCRA 28.6%) and VKA + DAPT (SUCRA 29.3%) showed the highest safety ranking. On the contrary, SAPT (SUCRA 92.3%), DAPT (SUCRA 61.9%) showed the lowest safety rankings, because they had the highest possibility of myocardial infarction.

### 3.5. Effect on Stroke Event of Different Treatments

The results concluding 21 trials [12,13,15,16,17,19,21,22,23,24,25,26,27,28,29,30,31,33,35,36,41] of 87,715 patients were demonstrated. In Figure 4d, VKA significantly reduced the rate of stroke compared with SAPT (OR = 3.45, 95% CI = 1.17–10.18). The results indicated that the SUCRA curve (91.2%) of SAPT was highest in those eight different treatments on ACS. VKA group showed the lowest SUCRA curve (7.1%), which was much better than other treatments.

### 3.6. Effect on Death of Different Treatments

A total of 26 trials, involving 95,493 people, were conducted to assess the impact of anticoagulant therapy and antiplatelet therapy on mortality. The studies referenced are numbered [12,13,15,16,17,18,19,21,23,24,25,26,27,28,29,30,31,32,33,35,36,37,38,40,41]. According to Figure 4e, the eight treatments did not exhibit any significant differences. However, SAPT (SUCRA 66.6%) was found to be the least effective in preventing death. The VKA treatment, with a SUCRA value of 37.7%, demonstrated the highest level of effectiveness.

### 3.7. Risks of Bias

**Figure 5:**
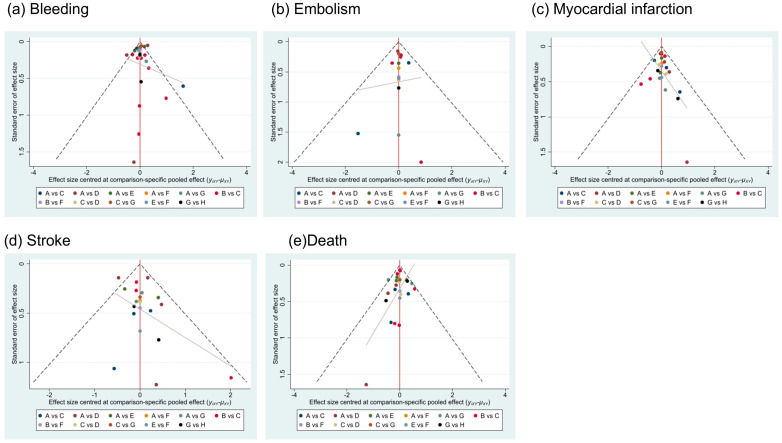
Publication bias. A = NOAC + SAPT (non-vitamin K antagonist oral anticoagulants plus single-agent platelet inhibitor therapy), B = NOAC + DAPT (non-vitamin K antagonist oral anticoagulants plus dual antiplatelet therapy), C = DAPT (dual antiplatelet therapy), D = SAPT (single-agent platelet inhibitor therapy), E = VKA + SAPT (vitamin K antagonists plus single-agent platelet inhibitor therapy), F = VKA + DAPT (vitamin K antagonists plus dual antiplatelet therapy), G = NOAC (non-vitamin K antagonist oral anticoagulants), H = VKA (vitamin K antagonists).

The results of publication bias in studies contributing to primary outcomes were displayed in Figure 5, and risks of bias in bleeding, myocardial infarction, embolism and death were low. The scatter plot distribution is relatively concentrated. Risk of bias in stroke was high because there were various studies with small sample sizes included. We performed a meta-regression analysis to assess the association between follow-up duration and stroke risk, and the results demonstrated that follow-up time was not a significant source of heterogeneity for stroke outcomes.

## 4. Discussion

This network meta-analysis compared different anticoagulants, antiplatelets, and combinations of both therapies in patients with ACS. The five complications we analyzed in this meta-analysis revealed that NOAC + SAPT could significantly reduce complications and play an important role in treatment effect. However, SAPT showed the lowest effectiveness or safety among these therapies. Although VKA showed the lowest rate of myocardial infarction, stroke, and death, it could not show safety on bleeding. Other treatments showed no significant difference. Overall, our results concluded that NOAC + SAPT shows more safety and effectiveness in the treatment of ACS, which provides new evidence for this field.

Antiplatelet and anticoagulant drugs are central to ACS and post-PCI treatment but increase bleeding risk [42,43]. Balancing bleeding and embolism risks remains a key challenge [44]. Our meta-analysis showed that single antiplatelet therapy (aspirin or P2Y12 inhibitors) in ACS has the highest embolism risk and poorest efficacy, while NOAC monotherapy carries a notable myocardial infarction risk. Although NOACs are generally safer and easier to use than VKAs [45,46], we found no clear benefit over VKAs in ACS, aligning with previous studies [47]. Combination therapies are more effective when monotherapy is insufficient [48]. DAPT lowers bleeding risk but shows considerable embolism and stroke risk, limiting its benefit in ACS. In contrast, VKA or NOAC combined with single antiplatelet therapy offers better thrombosis prevention with higher bleeding risk, but no difference was found between these two combinations. Overall, double antithrombotic therapy is more effective and safer than monotherapy in ACS.

Despite the elevated bleeding risk associated with triple therapy in post-PCI atrial fibrillation patients, European guidelines continue to endorse this approach when the net clinical benefit is favorable [49]. Research by Davide’s team revealed that NOAC plus P2Y12 inhibitor dual therapy had a more favorable safety profile than triple therapy approaches [50]. Toshiki’s meta-analysis showed the risk of stent thrombosis was not significantly different in double antithrombotic therapy vs. triple antithrombotic therapy [51]. Our net meta-analysis analyzed and compared VKA + DAPT and NOAC + DAPT on safety in ACS. NOAC + DAPT trended toward lower rates of stroke compared with VKA + DAPT but did not reach a significantly statistical difference. NOAC + DAPT significantly reduced rate of bleeding compared with VKA + DAPT. Our net meta-analysis indicated that NOAC + DAPT may be a better choice in triple antithrombotic therapy [52,53,54]. Nevertheless, further research is required to determine the effectiveness and safety of VKA + DAPT and NOAC + DAPT. In our analysis, bleeding outcomes were extracted based on the definitions used in the original trials (e.g., TIMI, BARC, GUSTO). Most of the included studies clearly distinguished between major and minor bleeding. However, due to variability in definitions and incomplete reporting across studies, a consistent grading system could not be uniformly applied.

When comparing double antithrombotic therapy strategies to triple antithrombotic therapy, it was shown that the risk of bleeding increased with triple antithrombotic therapy, particularly in the combination of VKA and DAPT. In trials that reported an elevated bleeding risk associated with the combination of VKA and DAPT, the bleeding events were predominantly major, as defined by BARC ≥ 3, TIMI major bleeding, or bleeding requiring transfusion or hospitalization. The observed increase in clinically significant major bleeding with this regimen suggests that its use should generally be avoided. Thus, alternative regimens with lower bleeding risk, such as NOAC-based strategies, are often preferred in contemporary practice to optimize safety without compromising efficacy. Significantly, in this comprehensive meta-analysis, there was minimal or nonexistent variation in the majority of outcomes, with the exception of bleeding. In addition, few research studies compared the safety of triple antithrombotic therapy with anticoagulant or antithrombotic monotherapy. In our net meta-analysis, comparing SAPT, NOAC + DAPT, VKA + DAPT, and NOAC + SAPT resulted in a significantly considerable risk of bleeding. The combination of anticoagulant and antiplatelet therapy was more effective than antiplatelet or anticoagulant therapy alone [7]. Moreover, NOAC + SAPT showed a statistically significant low risk of embolism and myocardial infarction. Of course, the above data demonstrated that combined antithrombotic therapy has certain advantages in patients who need long-term antithrombotic therapy [55]. Triple antithrombotic therapy, which combines anticoagulants with dual antiplatelet therapy, can significantly enhance antithrombotic efficacy but is also associated with a substantially increased risk of bleeding. In patients with ACS, the use of triple therapy should be strictly limited to high-risk individuals with a clear indication for anticoagulation, particularly those with atrial fibrillation, mechanical heart valves, or a history of thromboembolic events. Current mainstream strategies recommend using triple therapy only for a short duration (1 week to 1 month after PCI), followed by de-escalation to dual therapy consisting of an oral anticoagulant and a single P2Y12 inhibitor, aiming to balance thrombosis prevention with bleeding risk [49]. Treatment decisions should be individualized, taking into account the patient’s bleeding risk, thrombotic risk, and renal function. Overall, triple therapy should be used cautiously and reserved for selected patients with a clearly demonstrated benefit, with ongoing clinical monitoring and dynamic adjustments to the regimen.

In our network meta-analysis, heterogeneity in stroke outcomes was notable. We conducted a meta-regression to examine the effect of follow-up duration on stroke outcomes. The results showed no significant association, indicating that follow-up time did not contribute to heterogeneity in stroke risk. Therefore, we speculate that this could be partially attributed to the inclusion of several small-sized trials, increasing random variability and potential bias. Moreover, inconsistencies in stroke definitions and adjudication across studies may have contributed to clinical heterogeneity. Differences in patient baseline characteristics, such as atrial fibrillation prevalence, age, and comorbidities, further complicated the comparability of stroke risk. Additionally, different antithrombotic regimens (e.g., NOACs, VKAs, antiplatelets, or combinations) have varied mechanisms of action and safety profiles. Dabigatran, for instance, is known to increase gastrointestinal bleeding (GIB) risk [56], which may indirectly affect stroke outcomes through altered medication adherence or discontinuation. Taken together, these factors highlight the importance of cautious interpretation of stroke outcomes within indirect comparisons, particularly when data quality or event frequency is limited.

There is a wide variety of antiplatelet agents available. According to the latest guidelines, dual antiplatelet therapy consisting of aspirin combined with a P2Y12 inhibitor is recommended. Ticagrelor or prasugrel is the preferred first-line P2Y12 inhibitor. If these agents are contraindicated, not tolerated, unaffordable, or unavailable, clopidogrel may be used as an alternative option [57]. For patients at high risk of bleeding, current guidelines recommend several strategies. In those with a risk of gastrointestinal bleeding, the use of proton pump inhibitors (PPIs) is advised. Among patients receiving DAPT with ticagrelor, transitioning to ticagrelor monotherapy after at least 1 month post-PCI is suggested. Additionally, in patients requiring long-term oral anticoagulation, it is recommended to discontinue aspirin 1 to 4 weeks after PCI and continue with a P2Y12 inhibitor—preferably clopidogrel [58]. When antiplatelet and/or anticoagulant therapy has to be discontinued due to serious bleeding, clinicians are often compelled to resume anticoagulation therapy after a temporary interruption due to GIB. A meta-analysis suggests that the optimal timing for restarting NOACs is between 15 and 30 days after the GIB event [59]. For patients with a history of intracranial hemorrhage, the resumption of antithrombotic therapy may need to be delayed for 4 to 6 weeks or longer. In individuals previously treated with VKAs or NOACs should be preferred over VKAs upon reinitiation unless contraindicated. Among NOACs, apixaban or edoxaban may be especially suitable for elderly patients or those at high risk of gastrointestinal bleeding [60]. It is important to note that before resuming anticoagulation, patients should be receiving appropriate supportive treatments such as proton pump inhibitors, blood pressure control, and avoidance of NSAIDs. In addition, the risk of recurrent bleeding should be closely monitored [61].

In the context of ACS, transition strategies for anticoagulation require careful planning. When switching from parenteral anticoagulants, such as unfractionated heparin or bivalirudin to oral agents, the approach depends on the specific type of oral anticoagulant being initiated [62]. VKAs typically require an overlap period with parenteral anticoagulants until the target INR is achieved. Once adequate anticoagulation is established, NOACs can usually be started 12 to 24 h after discontinuation of the parenteral agents [63]. Conversely, in situations requiring reversal of oral anticoagulants, such as major bleeding or urgent surgery, parenteral anticoagulants can be reintroduced once bleeding is controlled and the clinical condition is stable. The timing and dosage should be individualized based on bleeding risk, thrombotic risk, and renal function [64]. A multidisciplinary approach involving cardiology, hematology, and critical care is often essential to optimize these transitions. For long-term anticoagulation management in patients with coronary artery disease who may require recurrent hospitalizations, interventions, or surgical revascularization, general ACS guidelines recommend aspirin plus a P2Y12 receptor inhibitor for 12 months, followed by aspirin monotherapy. For patients at high bleeding risk, DAPT can be shortened to 3 to 6 months, followed by single antiplatelet therapy. For those with high ischemic risk but low bleeding risk, extended DAPT or low-dose rivaroxaban combined with aspirin may be considered [65].

Although our meta-analysis provides comparative estimates of efficacy and safety, its applicability to high-risk subpopulations, such as the elderly, patients with chronic kidney disease, or those with a prior bleeding history, requires caution. The SWEDEHEART trial evaluated the efficacy and bleeding risk of ticagrelor versus clopidogrel in elderly patients aged ≥ 75 years. The results indicated that ticagrelor was associated with a significantly increased risk of bleeding in this population, while the reduction in ischemic events was limited [66]. The POPular AGE trial showed that clopidogrel significantly reduced bleeding events without a notable difference in ischemic protection. These findings suggest that elderly patients may benefit more from a conservative antiplatelet strategy, and potent antiplatelet agents should be used with caution in this population [67]. The CREST Study indicated that ACS patients with an eGFR < 60 mL/min face elevated risks of both bleeding and major adverse cardiovascular events (MACE) during DAPT, highlighting the need for individualized risk assessment in this population [68]. These groups are frequently encountered in clinical settings but are often underrepresented in randomized controlled trials. Given their increased susceptibility to both ischemic and hemorrhagic complications, individualized antithrombotic strategies may be warranted. Further research leveraging real-world data or patient-level meta-analyses is needed to optimize care in these vulnerable populations.

This meta-analysis has several important limitations that should be acknowledged. First, by focusing on eight specific therapies and excluding heparin or placebo-controlled trials, the strength of our network analysis conclusions may be limited. Second, insufficient primary data prevented subgroup analyses by treatment stages, leaving uncertainty about therapy effects on complications and mortality in ACS patients. Third, many treatment combinations lacked direct comparisons, requiring reliance on indirect effect estimates. Additionally, variability in outcome definitions, follow-up durations, and adjudication criteria across studies may have influenced results. Our classification of drugs by mechanism rather than individual agents may obscure important safety profile differences. A notable limitation is the uncertain duration of triple therapy (NOAC + DAPT), as most protocols transition to dual therapy within 1–2 weeks, yet precise timing data were unavailable. Fourth, our analysis does not actually differentiate between bare-metal stents and drug-eluting stents, despite their known impact on antithrombotic therapy selection and duration in ACS and PCI patients. Therefore, further prospective studies are warranted to evaluate the safety and efficacy of various antithrombotic strategies across different stent types. Finally, while VKA and NOAC showed numerically lower risks of embolism, death, and MI compared to SAPT, these differences were not statistically significant. These limitations underscore the need for standardized outcome reporting, direct comparative studies, and larger-scale investigations to better characterize the efficacy and safety of antithrombotic therapies in ACS patients.

## 5. Conclusions

This network meta-analysis compared the safety of eight different antithrombotic therapies in patients with ACS. A strategy of dual therapy, especially with NOAC + SAPT, should be a reasonable alternative to triple therapy or monotherapy in patients with ACS indication for chronic anticoagulation. However, future studies are needed to judge the safety and effect of different antithrombotic therapies, due to their drawbacks on other complications.

## Figures and Tables

**Table 1:** Basic information of the induced studies.

First Author	Year	N	Type	Study	Intervention	Control	Timing of the Primary Endpoint	Endpoint	Diseases
Marco J.D. Tangelder [12]	2007	1883	RCT	ESTEEM	Ximelagatran (24, 36, 48, or 60 mg BID) plus aspirin (70 mg or 160 mg daily)	Aspirin (70 mg or 160 mg daily)	6-month	Death, myocardial infarction, and stroke	Acute coronary syndrome with atrial fibrillation
Alexander J.H. s [13]	2008	1715	RCT	APPRAISE	Apixaban (2.5 mg or 10 mg daily) plus aspirin (165 mg daily) or clopidogrel (75 mg daily)	Aspirin (165 mg daily) or clopidogrel (75 mg daily)	6-month	Bleeding, death, myocardial infarction, severe recurrent ischemia, and ischemic stroke	Acute coronary syndrome
J L Mega [14]	2009	3576	RCT	ATLAS ACS-TIMI 46	Rivaroxaban (5, 10, 15, and 20 mg daily) plus aspirin (75–100 mg daily)	Aspirin (75–100 mg daily)	6-month	TIMI major or minor bleeding, death, myocardial infarction, stroke, and severe recurrent ischemia.	Acute coronary syndrome
Marc S Sabatine [15]	2009	3241	RCT	SEPIA-ACS1 TIMI 42	Otamixaban (0.08 mg/kg daily) plus aspirin or clopidogrel	Aspirin plus clopidogrel	6-month	Death, myocardial infarction, urgent revascularization, and TIMI major or minor bleeding.	Non-ST-elevation acute coronary syndromes
Jonas Oldgren [16]	2011	1861	RCT	RE-DEEM	Dabigatran (75, 110, or 150 mg daily) plus aspirin (100 mg daily) and clopidogrel (75 mg daily)	Aspirin (100 mg daily) and clopidogrel (75 mg daily)	6-month	All bleeds, deaths, myocardial infarction, severe recurrent ischemia, and stroke	Acute coronary syndromes
John H. Alexander [17]	2011	7392	RCT	APPRAISE-2	Apixaban (5 to 20 mg daily) plus aspirin and clopidogrel	Aspirin and clopidogrel	8-month	Cardiovascular death, myocardial infarction, ischemic stroke, and TIMI bleeding	Acute coronary syndrome
Gabriel Steg [18]	2011	1279	RCT	RUBY-1	Darexaban (30 and 60 mg daily) plus aspirin 75–325 mg and clopidogrel (75 mg daily)	Aspirin 75–325 mg and Clopidogrel (75 mg daily)	28-week	Major and clinically relevant non-major (CRNM) bleeding, death, myocardial infarction, non-fatal stroke, and severe recurrent ischemia	Acute coronary syndrome including primary percutaneous coronary intervention (PCI), thrombolysis, or medical management
Jessica L. Mega [19]	2012	15,526	RCT	ATLAS ACS 2–TIMI 51	Rivaroxaban (2.5 mg twice-daily) pius aspirin (70 mg daily)	Aspirin (70 mg daily)	12-week	Death, myocardial infarction, stroke, stent thrombosis, and TIMI major bleeding	Acute coronary syndrome
Hisao Ogawa [20]	2013	151	RCT	APPRAISE-J	Apixaban (2.5 mg twice daily) plus aspirin (100 mg daily)	Aspirin (100 mg daily)	6-month	ISTH and CRNM bleeding, deaths, non-fatal myocardial infarction, and stroke	Acute coronary syndrome
Philippe Gabriel Steg [21]	2013	13,229	RCT	TAO	Otamixaban (0.08 mg/kg daily)	Aspirin plus clopidogrel, prasugrel or ticagrelor	1-month	ISTH and CRNM bleeding, death, myocardial infarction, and any stroke	Non-ST-Segment elevation acute coronary syndromes
Willem J M Dewilde [22]	2013	563	RCT	WOEST	Oral anticoagulation plus clopidogrel (75 mg daily) and aspirin (80–100 mg daily)	Oral anticoagulation plus clopidogrel (75 mg daily)	12-month	Any bleeding event, death, myocardial infarction, target-vessel revascularization, stroke, and stent thrombosis	Acute coronary syndrome undergoing percutaneous coronary intervention
C. Michael Gibson [23]	2016	2124	RCT	PIONEER AF-PCI	Rivaroxaban (10 mg daily) plus aspirin (75–100 mg daily) and clopidogrel (75 mg once daily)	Vitamin K antagonist (once daily) plus aspirin (75–100 mg per day) and clopidogrel (75 mg daily)	12-month	Death from cardiovascular causes, myocardial infarction, and stroke	Acute coronary syndrome with atrial fibrillation undergoing PCI
Haiyan Xu [24]	2016	14,977	RCT	ENGAGE AF-TIMI 48	Edoxaban (30 or 60 mg daily) plus aspirin (100 mg daily)	Warfarin plus aspirin (100 mg daily)	3-month	Major bleeding, all-cause death, stroke, and systemic embolic events	Acute coronary syndrome with atrial fibrillation
E Magnus Ohman [25]	2017	3037	RCT	GEMINI-ACS-1	Rivaroxaban (2.5 mg twice daily) plus aspirin (100 mg daily) and clopidogrel (75 mg daily) or ticagrelor (90 mg twice daily)	Aspirin and clopidogrel (75 mg daily) or ticagrelor (90 mg twice daily)	180-day	TIMI bleeding, death, myocardial infarction, stroke, and stent thrombosis.	Acute coronary syndrome
J.W. Eikelboom [26]	2017	18,278	RCT	COMPASS	Rivaroxaban (5 mg twice daily) plus aspirin (100 mg once daily)	Aspirin (100 mg once daily)	6-month	All bleeding, death, myocardial infarction, stent thrombosis, and stroke	Acute coronary syndrome
Christopher P [27]	2017	2725	RCT	RE-DUAL PCI	Dabigatran (110 mg or 150 mg twice daily) plus clopidogrel (75 mg daily) or ticagrelor (90 mg twice daily) and aspirin (100 mg daily)	Warfarin plus clopidogrel (75 mg daily) or ticagrelor (90 mg twice daily) and aspirin (100 mg daily)	3-month	ISTH and CRNM bleeding, death, myocardial infarction, definite stent thrombosis, and any stroke	Acute coronary syndrome with atrial fibrillation who had undergone PCI
David Kopin [28]	2017	18,201	RCT	ARISTOTLE	Apixaban (5 mg twice daily)	Warfarin (maintaining an international normalized ratio of 2.0–3.0)	1.8-year	Stroke or systemic embolism, myocardial infarction, all-cause mortality, major bleeding and clinically relevant non-major bleeding	Acute coronary syndrome with atrial fibrillation
Faiez Zannad [29]	2018	5022	RCT	COMMANDER HF	Rivaroxaban (2.5 mg twice daily) plus aspirin (100 mg daily) and a P2Y12-receptor antagonist	Aspirin (100 mg daily) plus P2Y12-receptor antagonist	3-month	Death from any cause, myocardial infarction, any bleeding, and stroke.	Acute coronary syndrome
Sean T. Chen [30]	2018	655	RCT	ROCKET AF	Rivaroxaban (20 mg once daily)	Warfarin (dose-adjusted warfarin with a target maintaining an international normalized ratio of 2.5)	4-month	Major or NMCR bleeding, major bleeding, intracranial hemorrhage, and hemorrhagic stroke	Acute coronary syndrome with atrial fibrillation
Renato D. Lopes [31]	2019	4614	RCT	AUGUSTUS	Apixaban (5 mg twice daily) plus aspirin (100 mg daily)	Aspirin (100 mg daily)	6-month	Major or clinically relevant non-major bleeding, stroke, myocardial infarction, stent thrombosis, and urgent revascularization	Acute coronary syndrome or PCI in atrial fibrillation
Pascal Vranckx [32]	2019	1506	RCT	ENTRUST-AF PCI	Edoxaban (60 mg once daily) plus a P2Y12 inhibitor	Warfarin plus P2Y12 inhibitor and aspirin (100 mg once daily)	12-month	Major or clinically relevant non-major (CRNM) bleeding, cardiovascular death, stroke, systemic embolic events, and definite stent thrombosis	Acute coronary syndrome with atrial fibrillation
Yukiko Matsumura-Nakano [33]	2019	680	RCT	OAC-ALONE	Warfarin or NOAC (dabigatran 150 or 110 mg twice daily, rivaroxaban 15 or 10 mg once daily, apixaban 5 or 2.5 mg twice daily, and edoxaban 60 or 30 mg once daily) plus clopidogrel (75 mg daily) or aspirin (81–324 mg daily)	Clopidogrel (75 mg daily) or aspirin (81–324 mg daily)	1-year	All-cause death, myocardial infarction, stroke, and systemic embolism	Acute coronary syndrome with atrial fibrillation
Francesco Franchi [34]	2019	75	RCT	EDOX-APT	Edoxaban (60 mg once daily) plus aspirin and clopidogrel	Aspirin and clopidogrel	3-month	Any bleeding event	Acute coronary syndrome
Hidehira Fukaya [35]	2020	1075	RCT	AFIRE	Rivaroxaban (10 mg once daily) plus P2Y12 inhibitor or aspirin (100 mg once daily)	Rivaroxaban (10 mg once daily)	24.1-month	Non-major bleeding and any bleeding events, stroke, systemic embolism, myocardial infarction, unstable angina requiring revascularization, and death from any cause	Acute coronary syndrome with atrial fibrillation
Xinbing Liu [36]	2021	106	RCT	Xinbing Liu	Ticagrelor (90 mg twice daily) plus rivaroxaban (15 mg once daily)	Aspirin (100 mg once daily) and clopidogrel (75 mg once daily) plus warfarin (once daily)	1-year	Cardiovascular causes, myocardial infarction, stroke, stent thrombosis, and clinically significant bleeding	Acute coronary syndrome undergoing with nonvalvular atrial fibrillation (NVAF) undergoing percutaneous coronary intervention (PCI)
Zhongfan Zhang [37]	2022	279	RCT	Zhongfan Zhang	Rivaroxaban (2.5 mg twice daily) plus dual antiplatelet therapy (DAPT) (low-dose aspirin 100 mg daily therapy and clopidogrel 75 mg daily or ticagrelor 90 mg twice daily)	Dual antiplatelet therapy (DAPT) (low-dose aspirin 100 mg daily therapy and clopidogrel 75 mg daily or ticagrelor 90 mg twice daily)	180-day	All-cause mortality, systemic embolism, rehospitalization for cardiovascular events, and bleeding	Anterior ST-segment elevation myocardial infarction
Marco Valgimigli [38]	2022	4579	RCT	MASTER DAPT	Aspirin (75 mg daily) plus P2Y12 inhibitor including clopidogrel (75 mg daily), prasugrel (10 mg/day or 5 mg/day), or Ticagrelor (180 mg/day)	Aspirin (75 mg daily)	11-month	Major or clinically relevant non-major bleeding	Acute coronary syndrome undergoing percutaneous coronary intervention
Prapa Kanagaratnam [39]	2023	3602	RCT	C19-ACS	NOAC (rivaroxaban 2.5 mg twice daily) plus DAPT (aspirin 75 mg once daily and clopidogrel 75 mg once daily)	DAPT (aspirin 75 mg once daily and clopidogrel 75 mg once daily)	30-day	Bleeding and death	COVID-19 acute coronary syndrome trial
Ying X Gue [40]	2024	120	RCT	VaLiDate-R	Clopidogrel (75 mg daily) plus rivaroxaban (2.5 mg twice daily)	Clopidogrel (75 mg daily)	6-month	Myocardial infarction, stroke or cardiovascular death, and bleeding events	Acute coronary syndrome (including those with STEMI, NSTE-ACS and unstable angina)
Zhen Ge [41]	2024	3400	RCT	ULTIMATE-DAPT	Ticagrelor (90 mg twice daily) plus oral aspirin (100 mg once daily)	Ticagrelor (90 mg twice daily) plus a matching oral placebo	11-month	All-cause death, myocardial infarction, stroke, stent thrombosis, or clinically relevant bleeding	Acute coronary syndrome after percutaneous coronary intervention

**Table 2:** Absolute event rates of different outcomes.

Study	Intervention	Control	Absolute Event Rates of Bleeding		Absolute Event Rates of Embolism		Absolute Event Rates of Death		Absolute Event Rates of Myocardial Infarction		Absolute Event Rates of Stroke	
			Intervention	Control	Intervention	Control	Intervention	Control	Intervention	Control	Intervention	Control
ESTEEM	NOAC + SAPT	SAPT	-	-	-	-	3.1%	3.3%	4.3%	5.6%	0.9%	2.0%
APPRAISE	NOAC + SAPT	SAPT	21.6%	10.5%	-	-	2.5%	2.0%	2.0%	3.3%	0.2%	0.3%
ATLAS ACS-TIMI 46	NOAC + SAPT	SAPT	11.3%	3.2%	-	-	-	-	-	-	-	-
SEPIA-ACS1 TIMI 42	NOAC + SAPT	DAPT	27.3%	21.9%	-	-	1.2%	1.8%	2.3%	3.1%	0.3%	0.2%
RE-DEEM	NOAC + DAPT	DAPT	13.2%	6.5%	-	-	2.1%	3.8%	2.1%	1.1%	0.1%	0.8%
APPRAISE-2	NOAC + DAPT	DAPT	3.2%	1.2%	0.9%	1.3%	4.2%	3.9%	4.9%	5.3%	0.6%	0.9%
RUBY-1	NOAC + DAPT	DAPT	14.5%	8.2%	0.0%	0.0%	0.7%	0.6%	2.7%	1.9%	-	-
ATLAS ACS 2–TIMI 51	NOAC + SAPT	SAPT	13.8%	6.6%	1.0%	1.4%	6.1%	7.4%	3.8%	4.5%	1.6%	1.5%
APPRAISE-J	NOAC + DAPT	DAPT	41.8%	33.3%	-	-	-	-	-	-	-	-
TAO	NOAC	DAPT	11.9%	5.6%	0.9%	1.1%	1.0%	0.9%	4.7%	5.0%	0.4%	0.3%
WOEST	VKA + SAPT	VKA + DAPT	19.4%	44.4%	1.4%	3.2%	2.5%	6.3%	3.2%	4.6%	1.1%	2.8%
PIONEER AF-PCI	NOAC + DAPT	VKA + DAPT	16.1%	24.0%	0.8%	0.6%	2.1%	1.6%	2.6%	3.0%	1.3%	1.0%
ENGAGE AF-TIMI 48	NOAC + SAPT	VKA + SAPT	13.4%	18.1%	-	-	2.6%	3.6%	0.9%	0.9%	2.0%	1.9%
GEMINI-ACS-1	NOAC + SAPT	DAPT	24.6%	20.9%	1.1%	1.1%	1.3%	1.1%	3.7%	3.2%	0.5%	0.8%
COMPASS	NOAC + SAPT	SAPT	12.3%	7.4%	0.3%	0.4%	3.4%	4.1%	1.9%	2.2%	0.9%	1.6%
RE-DUAL PCI	NOAC + SAPT	VKA + DAPT	27.1%	42.9%	1.5%	0.8%	5.6%	4.9%	4.5%	3.0%	1.7%	1.3%
ARISTOTLE	NOAC	VKA	4.6%	4.3%	-	-	7.9%	4.3%	2.0%	3.0%	2.6%	1.8%
COMMANDER HF	NOAC + DAPT	DAPT	3.3%	2.0%	0.6%	0.6%	21.8%	22.1%	3.9%	4.7%	2.0%	3.0%
ROCKET AF	NOAC	VKA	27.6%	25.1%	1.3%	0.9%	14.3%	16.3%	6.3%	4.7%	5.7%	2.4%
AUGUSTUS	NOAC + SAPT	VKA + SAPT	24.1%	35.5%	0.6%	0.8%	3.3%	3.2%	3.1%	3.5%	0.6%	1.1%
ENTRUST-AF PCI	NOAC + SAPT	VKA + SAPT	23.0%	26.5%	-	-	6.5%	6.1%	-	-	-	-
OAC-ALONE	NOAC + SAPT	NOAC	10.4%	7.8%	0.0%	0.6%	9.0%	11.6%	1.2%	2.3%	5.2%	3.5%
EDOX-APT	NOAC + DAPT	DAPT	8.0%	4.0%	-	-	-	-	-	-	-	-
AFIRE	NOAC + SAPT	NOAC	21.6%	13.2%	-	-	6.6%	3.7%	0.7%	1.2%	2.5%	1.9%
Xinbing Liu	NOAC + SAPT	DAPT	7.4%	26.9%	5.6%	0.0%	7.4%	5.8%	7.4%	15.4%	16.7%	19.2%
Zhongfan Zhang	NOAC + DAPT	DAPT	2.9%	1.4%	2.2%	5.0%	2.2%	2.1%	-	-	-	-
MASTER DAPT	DAPT	SAPT	4.8%	2.2%	-	-	1.8%	1.6%	1.2%	1.5%	-	-
C19-ACS	NOAC + DAPT	DAPT	1.9%	2.5%	-	-	-	-	-	-	-	-
VaLiDate-R	NOAC + SAPT	SAPT	1.6%	0.0%	-	-	1.6%	0.0%	0.0%	1.7%	-	-
ULTIMATE-DAPT	DAPT	SAPT	4.6%	2.1%	0.3%	0.3%	0.8%	0.7%	0.6%	1.0%	0.6%	1.0%

## Data Availability

The following information was supplied regarding data availability: This is a systematic review/meta-analysis. All data generated or analyzed during this study are included in Supplementary Information Files.

## Supplementary Materials

The supporting information can be downloaded at: https://www.mdpi.com/article/10.3390/biomedicines13082027/s1. References [3,8,48,69,70,71,72] are cited in the supplementary materials.
