# Supplementary material for: Comparative Safety of Anticoagulant, Antiplatelet and the Combination of Both for Acute Coronary Syndrome: A Systematic Review and Network Meta-Analysis
Source: Biomedicines. 2025 Aug 20;13(8):2027. doi: 10.3390/biomedicines13082027 (PMC12383640; doi:10.3390/biomedicines13082027)
Supplement: Supplementary file 1 [file biomedicines-13-02027-s001.zip › CRD42024542826.pdf]

Comparative safety of anticoagulant, antiplatelet and combine both for acute coronary syndrome: asystematic review and network meta-analysis

To enable PROSPERO to focus on COVID-19 submissions, this registration record has undergone basic automated checks for eligibility and is published exactly as submitted. PROSPERO has never provided peer review, and usual checking by the PROSPERO team does not endorse content. Therefore, automatically published records should be treated as any other PROSPERO registration. Further detail is provided here.

Citation

Qingsheng Niu, Ziyi Zhu. Comparative safety of anticoagulant, antiplatelet and combine both for acute coronary syndrome: asystematic review and network meta-analysis. PROSPERO 2024 CRD42024542826 Available from: https://www.crd.york.ac.uk/prospero/display_record.php?ID=CRD42024542826

Review question

Antithrombotic therapy plays an important role in acute coronary syndrome (ACS). The combination of anticoagulant and antiplatelet therapy resulted in fewer complications and stronger potency compared to traditional monotherapy. Our net-meta aimed to compare and rank the safety of different treatments used in patients with acute coronary syndrome.

Searches

PubMed/MEADLINE, Cochrane/CENTRAL, and Scopus

Types of study to be included

RCTs and observational studies

Condition or domain being studied

Acute coronary syndrome arises from the rupture or erosion of unstable atherosclerotic plaques within the coronary arteries, leading to thrombus formation and subsequently causing an acute myocardial ischemic syndrome.

Participants/population

(1) randomized controlled trials and observational studies that compared anticoagulant, antiplatelet or combination therapy, (intervention group or control group) in patients with coronary heart disease; (2) patients were administered direct oral warfarin, anticoagulants, aspirin or P2Y12 receptor antagonists after coronary heart disease; (3) efficacy and safety endpoints.

Intervention(s), exposure(s)

Anticoagulant, antiplatelet and combine both

Comparator(s)/control

Eight types of interventions were included: VKA, NOAC, DAPT, SAPT, VKA+DAPT, VKA+SAPT, NOAC+DAPT, NOAC+SAPT.

Main outcome(s)

Bleeding, death, myocardial infarct, stroke, and stent embolism

Additional outcome(s)

Not applicable

Measures of effect

Risk ratio (OR) with confidence interval (CI) of 95% was adopted as a representative measure of dichotomous outcomes

Data extraction (selection and coding)

(1) Letters to the editor, reviews, and animal studies; (2) there was a combination of heparin or other nonantithrombotic interventions; and (3) the studies were duplicates

Risk of bias (quality) assessment

Assessment of risk of bias was done using RevMan 5.3.

Strategy for data synthesis

The rates of events with each antiplatelet, anticoagulant or combination treatment were entered as an individual study arm, and data were pooled in a multiple treatment NMA that allows integration of direct and indirect comparisons. Heterogeneity was also quantified using χ^2 tests and the inconsistency statistic (I^2). Heterogeneity was considered significant for values of P >0.1 and I^2< 50%. When a moderate or high heterogeneity (I^2 >50% and p-value <0.1) was observed, a random-effect model was employed; otherwise, a fixed-effect model was applied.

Analysis of subgroups or subsets

Our analysis is network meta analysis. We did not plan investigation of subgroups

Contact details for further information

Qingsheng Niu

1390095443@qq.com

Organisational affiliation of the review

West China Hospital of Sichuan University

Review team members and their organisational affiliations

Dr Qingsheng Niu. West China Hospital of Sichuan University

Ziyi Zhu. West China Hospital of Sichuan University

Type and method of review

Meta-analysis, Network meta-analysis, Systematic review

Anticipated or actual start date

01 April 2024

Anticipated completion date

01 June 2024

Funding sources/sponsors

Sichuan Provincial Cadre Health Research Projec

Grant number(s)

State the funder, grant or award number and the date of award

No. CGY 2022-117

Conflicts of interest

None known

Language

English

Country

China

Stage of review

Review Ongoing

Subject index terms status

Subject indexing assigned by CRD

Subject index terms

MeSH headings have not been applied to this record

Date of registration in PROSPERO

13 May 2024

Date of first submission

03 May 2024

Stage of review at time of this submission

Preliminary searches	No	No
Piloting of the study selection process	Yes	No
Formal screening of search results against eligibility criteria	Yes	No
Data extraction	No	No
Risk of bias (quality) assessment	No	No
Data analysis	No	No

The record owner confirms that the information they have supplied for this submission is accurate and complete and they understand that deliberate provision of inaccurate information or omission of data may be construed as scientific misconduct.

The record owner confirms that they will update the status of the review when it is completed and will add publication details in due course.

Versions

13 May 2024

13 May 2024
