# Supplementary material for: Comparative Safety of Anticoagulant, Antiplatelet and the Combination of Both for Acute Coronary Syndrome: A Systematic Review and Network Meta-Analysis
Source: Biomedicines. 2025 Aug 20;13(8):2027. doi: 10.3390/biomedicines13082027 (PMC12383640; doi:10.3390/biomedicines13082027)
Supplement: Supplementary file 1 [file biomedicines-13-02027-s001.zip › Rationale.pdf]

Systematic Review and/or Meta-Analysis Rationale

For systematic reviews / meta-analyses, authors need to provide the following information:

The rationale for conducting the systematic review / meta-analysis

Long-term antiplatelet and anticoagulant therapies play a key role in preventing complications associated with acute coronary syndrome. Oral antiplatelet therapy and anticoagulants are recommended therapeutic approaches in the initial management of acute coronary syndrome, regardless of whether the treatment is invasive or non-invasive (1). Multiple systematic reviews and meta-analyses have already demonstrated the potential efficacy and safety of anticoagulant plus antiplatelet therapy in acute coronary syndrome and stable coronary artery disease(2, 3). Although NOACs (non-vitamin K antagonist oral anticoagulant) plus antiplatelet were associated with a lower risk of all-cause mortality but increased the risk of major and nonmajor bleeding. However, many previous studies remain controversial and are limited by differences in antithrombotic therapies in patients with ACS(4). Furthermore, there have been additional studies published that should be included in a brand-new meta-analysis, necessitating an update of these results. As a result, our meta-analysis has updated new literature on antithrombotic therapy in patients with ACS. We classified antiplatelet therapy into single-agent platelet and dual-antiplatelet therapy, and we further divided into eight different antithrombotic treatments according to the mechanism and type of action of the drugs. In addition, more patients with acute coronary syndrome are included. We conducted this network meta-analysis to evaluate association between anticoagulant, antiplatelet and combine both for acute coronary syndrome and risk of bleeding, death, myocardial infarct, stroke, and stent embolism. We hope this meta-analysis can help us find the optimal antithrombotic treatment strategy to balance the risks of bleeding and embolism.

The contribution that it makes to knowledge in light of previously published related reports, including other meta-analyses and systematic reviews.

Kyriakos Dimitriadis's network meta-analysis of four antithrombotic strategies demonstrated that in patients with ACS the combination of dual antithrombotic therapy-based NOAC is associated with a significantly lower risk of major bleeding events(5). However, Abdulmajeed Alharbi showed that DOACs plus antiplatelet treatment (APT) for the prevention of left ventricular thrombus in patients with ACS were associated with a lower risk of all-cause mortality but increased the risk of major and nonmajor bleeding(6). Due to all the controversies and limitations of previous studies, the aim of our study was to conduct a comprehensive analysis of published studies to evaluate association between anticoagulant, antiplatelet and combine both for acute coronary syndrome and risk of bleeding, death, myocardial infarct, stroke, and stent embolism.

References

1. Bhatt DL, Hulot JS, Moliterno DJ, Harrington RA. Antiplatelet and Anticoagulation Therapy for Acute Coronary Syndromes. Circ Res. 2014;114(12):1929-43.

2. Liu LL, Lei H, Hu JH, Tang Y, Xu DY. Direct Oral Anticoagulants Combined with Antiplatelet Therapy in the Treatment of Coronary Heart Disease: An Updated Meta-analysis. Drugs. 2021;81(17):2003-16.

3. Lin YW, Cai ZG, Dong SH, Liu HD, Pang XL, Chen QL, et al. Comparative efficacy and safety of antiplatelet or anticoagulant therapy in patients with chronic coronary syndromes after percutaneous coronary intervention: A network meta-analysis of randomized controlled trials. Front Pharmacol. 2022;13.

4. Capodanno D, Di Maio M, Greco A, Bhatt DL, Gibson CM, Goette A, et al. Safety and Efficacy of Double Antithrombotic Therapy With Non-Vitamin K Antagonist Oral Anticoagulants in Patients With Atrial Fibrillation Undergoing Percutaneous Coronary Intervention: A Systematic Review and Meta-Analysis. J Am Heart Assoc. 2020;9(16).

5. Dimitriadis K, Soulaidopoulos S, Doundoulakis I, Iliakis P, Tsiachris D, Tsiou P, et al. A network meta-analysis of the antithrombotic strategies in patients with atrial fibrillation and percutaneous coronary interventions: Focus on bleeding. Hell J Cardiol. 2023;73:69-72.

6. Alharbi A, Mhanna M, Alyosif M, Pena C, Jabr A, Alsughayer A, et al. Safety and Efficacy of Direct Oral Anticoagulant in Addition to Antiplatelet Therapy After Acute Coronary Syndrome: A Systemic Review and Meta-analysis of 53,869 Patients. Clin Ther. 2024;46(1):e1-e6.
