# Supplementary material for: Comparative Safety of Anticoagulant, Antiplatelet and the Combination of Both for Acute Coronary Syndrome: A Systematic Review and Network Meta-Analysis
Source: Biomedicines. 2025 Aug 20;13(8):2027. doi: 10.3390/biomedicines13082027 (PMC12383640; doi:10.3390/biomedicines13082027)
Supplement: Supplementary file 1 [file biomedicines-13-02027-s001.zip › raw data/League Table.pdf]

(a) Bleeding

VKA+DAPT	0.57 (0.35,0.92)	0.47 (0.29,0.77)	0.37 (0.24,0.57)	0.35 (0.19,0.62)	0.31 (0.13,0.73)	0.29 (0.18,0.47)	0.15 (0.09,0.24)
1.77 (1.09,2.86)	NOAC+DAPT	0.84 (0.50,1.40)	0.65 (0.43,0.98)	0.61 (0.36,1.04)	0.55 (0.24,1.24)	0.52 (0.38,0.70)	0.26 (0.16,0.41)
2.11 (1.30,3.42)		VKA+SAPT	0.78 (0.55,1.11)	0.73 (0.43,1.26)	0.65 (0.29,1.49)	0.62 (0.39,0.97)	0.31 (0.20,0.49)
2.70 (1.75,4.18)	1.53 (1.02,2.30)		NOAC+SAPT	0.94 (0.62,1.43)	0.84 (0.39,1.78)	0.79 (0.58,1.08)	0.40 (0.29,0.53)
2.88 (1.60,5.18)				NOAC	0.89 (0.48,1.66)	0.84 (0.54,1.32)	0.42 (0.26,0.69)
3.23 (1.37,7.60)					VKA	0.94 (0.44,2.03)	0.47 (0.21,1.04)
3.43 (2.13,5.51)	1.94 (1.42,2.65)					DAPT	0.50 (0.35,0.72)
6.85 (4.11,11.39)	3.87 (2.46,6.10)	3.25 (2.06,5.11)	2.53 (1.88,3.40)	2.38 (1.45,3.89)		2.00 (1.39,2.87)	SAPT

(b) Embolism

SAPT	0.73 (0.41,1.29)	0.67 (0.52,0.86)	0.61 (0.31,1.20)	0.61 (0.31,1.21)	0.59 (0.31,1.12)	0.52 (0.26,1.04)	0.42 (0.08,2.21)
	DAPT	0.92 (0.54,1.57)	0.84 (0.38,1.87)	0.83 (0.56,1.23)	0.80 (0.56,1.15)	0.72 (0.34,1.51)	0.58 (0.12,2.75)
1.50 (1.16,1.94)		NOAC+SAPT	0.92 (0.49,1.72)	0.91 (0.47,1.76)	0.88 (0.48,1.62)	0.79 (0.41,1.49)	0.63 (0.12,3.28)
			VKA+SAPT	0.99 (0.41,2.40)	0.96 (0.41,2.22)	0.85 (0.40,1.84)	0.69 (0.12,3.95)
				NOAC	0.96 (0.57,1.64)	0.86 (0.38,1.98)	0.70 (0.15,3.14)
					NOAC+DAPT	0.89 (0.42,1.90)	0.72 (0.15,3.56)

						VKA+DAPT	0.81 (0.14,4.51)
							VKA

(c) Myocardial infarction

SAPT	0.85 (0.67,1.08)	0.85 (0.63,1.16)	0.82 (0.73,0.92)	0.81 (0.61,1.08)	0.77 (0.58,1.02)	0.73 (0.51,1.05)	0.68 (0.35,1.34)
	DAPT	1.00 (0.70,1.44)	0.97 (0.77,1.21)	0.96 (0.81,1.14)	0.91 (0.77,1.06)	0.86 (0.60,1.25)	0.80 (0.42,1.52)
		VKA+SAPT	0.96 (0.72,1.28)	0.95 (0.65,1.41)	0.90 (0.62,1.32)	0.86 (0.56,1.31)	0.80 (0.39,1.65)
1.22 (1.08,1.37)			NOAC+SAPT	0.99 (0.75,1.31)	0.94 (0.72,1.22)	0.89 (0.63,1.27)	0.83 (0.42,1.63)
				NOAC	0.95 (0.75,1.19)	0.90 (0.60,1.35)	0.84 (0.45,1.55)
					NOAC+DAPT	0.95 (0.66,1.38)	0.89 (0.46,1.71)
						VKA+DAPT	0.93 (0.45,1.94)
							VKA

(d) Stroke

SAPT	0.75 (0.42,1.31)	0.75 (0.38,1.49)	0.69 (0.46,1.03)	0.62 (0.33,1.18)	0.61 (0.28,1.31)	0.50 (0.25,1.01)	0.29 (0.10,0.85)
	DAPT	1.00 (0.48,2.10)	0.92 (0.56,1.53)	0.83 (0.45,1.52)	0.81 (0.39,1.71)	0.67 (0.42,1.08)	0.39 (0.13,1.12)
		VKA+SAPT	0.92 (0.53,1.60)	0.83 (0.38,1.77)	0.81 (0.36,1.82)	0.67 (0.29,1.52)	0.39 (0.12,1.23)
			NOAC+SAPT	0.90 (0.53,1.53)	0.88 (0.44,1.75)	0.73 (0.38,1.38)	0.42 (0.15,1.17)

				NOAC	0.98 (0.42,2.26)	0.81 (0.38,1.70)	0.47 (0.20,1.12)
					VKA+DAPT	0.83 (0.39,1.76)	0.48 (0.14,1.60)
						NOAC+DAPT	0.58 (0.18,1.82)
3.45 (1.17,10.18)							VKA

(e) Death

SAPT	0.98 (0.69,1.39)	0.95 (0.63,1.45)	0.94 (0.57,1.55)	0.91 (0.71,1.17)	0.88 (0.60,1.29)	0.87 (0.58,1.32)	0.84 (0.44,1.60)
	DAPT	0.98 (0.75,1.28)	0.96 (0.59,1.57)	0.94 (0.68,1.29)	0.91 (0.58,1.40)	0.89 (0.61,1.32)	0.86 (0.45,1.64)
		NOAC+DAPT	0.98 (0.59,1.64)	0.96 (0.65,1.42)	0.93 (0.57,1.51)	0.91 (0.58,1.45)	0.88 (0.44,1.75)
			VKA+DAPT	0.97 (0.63,1.50)	0.94 (0.57,1.55)	0.93 (0.54,1.59)	0.89 (0.42,1.89)
				NOAC+SAPT	0.97 (0.71,1.31)	0.95 (0.67,1.36)	0.92 (0.50,1.70)
					VKA+SAPT	0.99 (0.62,1.57)	0.95 (0.48,1.88)
						NOAC	0.96 (0.58,1.61)
							VKA
