# Supplementary material for: Comparative Safety of Anticoagulant, Antiplatelet and the Combination of Both for Acute Coronary Syndrome: A Systematic Review and Network Meta-Analysis
Source: Biomedicines. 2025 Aug 20;13(8):2027. doi: 10.3390/biomedicines13082027 (PMC12383640; doi:10.3390/biomedicines13082027)
Supplement: Supplementary file 1 [file biomedicines-13-02027-s001.zip › raw data/Software operation records of death.pdf]

```
__/   /   /___/   /   /___/       MP—Parallel Edition

 Statistics and Data Science       Copyright 1985-2021 StataCorp LLC
                                   StataCorp
                                   4905 Lakeway Drive
                                   College Station, Texas 77845 USA
                                   800-STATA-PC        https://www.stata.com
                                   979-696-4600        stata@stata.com

Stata license: Single-user 8-core , expiring  1 Jan 2025
Serial number: 501709301094
  Licensed to: 1
               1

Notes:
      1. Unicode is supported; see help unicode_advice.
      2. More than 2 billion observations are allowed; see help obs_advice.
      3. Maximum number of variables is set to 5,000; see help set_maxvar.

Running c:\ado\plus\profile.do ...

. use "E:\Meta 分析\重要数据\重新分析\重新分析的 RCT\死亡\死亡.dta"

. network setup r n, studyvar(id) trtvar(t) format(augment) or
Treatments used
   A (reference):                  1
   B:                              2
   C:                              3
   D:                              4
   E:                              5
   F:                              6
   G:                              7
   H:                              8

Measure                            Log odds ratio

Studies
   ID variable:                    id
   Number used:                        26
   IDs with zero cells:              38
   - count added to all their cells:   .5
   IDs with augmented reference arm:   3 5 8 11 12 14 21 23 32 36 37 39
   - observations added:               0.00001
   - mean in augmented observations:   study-specific mean
```

Network information
   Components:                     1 (connected)
   D.f. for inconsistency:         4
   D.f. for heterogeneity:         15

Current data
   Data format:                    augmented
   Design variable:                _design
   Estimate variables:             _y*
   Variance variables:             _S*
   Command to list the data:       list id _y* _S*, noo sepby(_design)

.
. network map
Graph command stored in F9

.
.
.
. set matsize 10000
set matsize ignored.
    Matrix sizes are no longer limited by c(matsize) in modern Statas.  Matrix sizes are now limited by edition of Stata.  See limits for more
    details.

.
. network meta i
Command is: mvmeta _y _S  , bscovariance(exch 0.5) longparm suppress(uv mm) eq(_y_B: des_BF, _y_D: des_CD, _y_F: des_EF, _y_G: des_CG) vars(_y_B _y_
> C _y_D _y_E _y_F _y_G _y_H)
Note: using method reml
Note: regressing _y_B on des_BF
Note: regressing _y_C on (nothing)
Note: regressing _y_D on des_CD
Note: regressing _y_E on (nothing)
Note: regressing _y_F on des_EF
Note: regressing _y_G on des_CG
Note: regressing _y_H on (nothing)
Note: 26 observations on 7 variables
Note: variance-covariance matrix is proportional to .5*I(7)+.5*J(7,7,1)

initial:       log likelihood = -121.67912
rescale:       log likelihood = -114.31922

```
rescale eq:    log likelihood = -112.76413
Iteration 0:   log likelihood = -112.76413
Iteration 1:   log likelihood = -111.94874  (not concave)
Iteration 2:   log likelihood = -111.87523
Iteration 3:   log likelihood = -111.68907
Iteration 4:   log likelihood = -111.63792  (not concave)
Iteration 5:   log likelihood = -111.60567  (not concave)
Iteration 6:   log likelihood = -111.60277
Iteration 7:   log likelihood =  -111.6027
Iteration 8:   log likelihood = -111.60268
Iteration 9:   log likelihood = -111.60268

Multivariate meta-analysis
Variance-covariance matrix = proportional .5*I(7)+.5*J(7,7,1)
Method = reml                              Number of dimensions    =     7
Restricted log likelihood = -111.60268         Number of observations  =    26
------------------------------------------------------------------------------
             | Coefficient  Std. err.      z    P>|z|     [95% conf. interval]
-------------+----------------------------------------------------------------
_y_B         |
      des_BF |   .0824591   .4811339     0.17   0.864     -.860546    1.025464
       _cons |   .0494004   .2504768     0.20   0.844    -.4415252     .540326
-------------+----------------------------------------------------------------
_y_C         |
       _cons |   .0617057   .2437057     0.25   0.800    -.4159487    .5393602
-------------+----------------------------------------------------------------
_y_D         |
      des_CD |  -.2435768   .3178728    -0.77   0.444    -.8665961    .3794424
       _cons |   .1843356   .0497643     3.70   0.000     .0867994    .2818717
-------------+----------------------------------------------------------------
_y_E         |
       _cons |   .0532334   .1098657     0.48   0.628    -.1620994    .2685661
-------------+----------------------------------------------------------------
_y_F         |
      des_EF |   1.163664   .5090048     2.29   0.022     .1660327    2.161295
       _cons |  -.1436631   .2028962    -0.71   0.479    -.5413324    .2540061
-------------+----------------------------------------------------------------
_y_G         |
      des_CG |    .511786   .3528353     1.45   0.147    -.1797585     1.20333
       _cons |  -.2600068   .1567587    -1.66   0.097    -.5672482    .0472346
-------------+----------------------------------------------------------------
_y_H         |
       _cons |   -.240202   .2536055    -0.95   0.344    -.7372596    .2568555
------------------------------------------------------------------------------
```

```
Estimated between-studies SDs and correlation matrix
--------------------------------------------------
           SD       _y_B       _y_C       _y_D       _y_E       _y_F       _y_G       _y_H
_y_B  .00001538         1          .          .          .          .          .          .
_y_C  .00001538        .5          1          .          .          .          .          .
_y_D  .00001538        .5         .5          1          .          .          .          .
_y_E  .00001538        .5         .5         .5          1          .          .          .
_y_F  .00001538        .5         .5         .5         .5          1          .          .
_y_G  .00001538        .5         .5         .5         .5         .5          1          .
_y_H  .00001538        .5         .5         .5         .5         .5         .5          1
--------------------------------------------------

Testing for inconsistency:
 ( 1)  [_y_B]des_BF = 0
 ( 2)  [_y_D]des_CD = 0
 ( 3)  [_y_G]des_CG = 0
 ( 4)  [_y_F]des_EF = 0

           chi2(  4) =    7.10
         Prob > chi2 =    0.254
mvmeta command stored as F9; test command stored as F8

.
. network meta c
Command is: mvmeta _y _S  , bscovariance(exch 0.5) longparm suppress(uv mm)  vars(_y_B
_y_C _y_D _y_E _y_F _y_G _y_H)
Note: using method reml
Note: using variables _y_B _y_C _y_D _y_E _y_F _y_G _y_H
Note: 26 observations on 7 variables
Note: variance-covariance matrix is proportional to .5*I(7)+.5*J(7,7,1)

initial:       log likelihood = -127.05915
rescale:       log likelihood = -116.48195
rescale eq:    log likelihood = -116.29585
Iteration 0:   log likelihood = -116.29585
Iteration 1:   log likelihood = -116.13428  (not concave)
Iteration 2:   log likelihood = -116.13391
Iteration 3:   log likelihood = -116.12991  (not concave)
Iteration 4:   log likelihood = -116.12936
Iteration 5:   log likelihood = -116.12927
Iteration 6:   log likelihood = -116.12926

Multivariate meta-analysis
```

```
Variance-covariance matrix = proportional .5*I(7)+.5*J(7,7,1)
Method = reml                          Number of dimensions    =     7
Restricted log likelihood = -116.12926        Number of observations  =    26
------------------------------------------------------------------------------
             | Coefficient  Std. err.      z    P>|z|     [95% conf. interval]
-------------+----------------------------------------------------------------
_y_B         |
       _cons |   .0435892   .2010505     0.22   0.828    -.3504625     .437641
-------------+----------------------------------------------------------------
_y_C         |
       _cons |   .0655091   .1629528     0.40   0.688    -.2538725    .3848906
-------------+----------------------------------------------------------------
_y_D         |
       _cons |   .0898048   .1257014     0.71   0.475    -.1565653    .3361749
-------------+----------------------------------------------------------------
_y_E         |
       _cons |  -.0332132   .1552363    -0.21   0.831    -.3374707    .2710444
-------------+----------------------------------------------------------------
_y_F         |
       _cons |   .0280817    .221679     0.13   0.899    -.4064011    .4625645
-------------+----------------------------------------------------------------
_y_G         |
       _cons |  -.0463673   .1797688    -0.26   0.796    -.3987077    .3059731
-------------+----------------------------------------------------------------
_y_H         |
       _cons |  -.0844433   .3150687    -0.27   0.789    -.7019666      .53308
------------------------------------------------------------------------------

Estimated between-studies SDs and correlation matrix
--------------------------------------------------
             SD        _y_B       _y_C       _y_D       _y_E       _y_F       _y_G       _y_H
_y_B  .19810219          1          .          .          .          .          .          .
_y_C  .19810219         .5          1          .          .          .          .          .
_y_D  .19810219         .5         .5          1          .          .          .          .
_y_E  .19810219         .5         .5         .5          1          .          .          .
_y_F  .19810219         .5         .5         .5         .5          1          .          .
_y_G  .19810219         .5         .5         .5         .5         .5          1          .
_y_H  .19810219         .5         .5         .5         .5         .5         .5          1
--------------------------------------------------
mvmeta command stored as F9

.
. network forest
```

. graph save "Graph" "E:\Meta 分析\重要数据\重新分析\重新分析的 RCT\死亡\1.gph"
file E:\Meta 分析\重要数据\重新分析\重新分析的 RCT\死亡\1.gph saved

. graph save "Graph" "E:\Meta 分析\重要数据\重新分析\重新分析的 RCT\死亡\2.gph"
file E:\Meta 分析\重要数据\重新分析\重新分析的 RCT\死亡\2.gph saved

. network rank max, all zero reps(5000) gen(prob)
Command is: mvmeta, noest pbest(max in 1, zero id(id) all reps(5000) gen(prob) stripprefix(_y_) zeroname(A) rename(A = 1, B = 2, C = 3, D = 4, E =
> 5, F = 6, G = 7, H = 8))

Estimated probabilities (%) of each treatment having each rank
- assuming the maximum parameter is the best
- using 5000 draws
- allowing for parameter uncertainty

```
---------------------------------------------------------
         |                 Treatment
    Rank |   1    2    3    4    5    6    7    8
----------+----------------------------------------------
    Best |  1.6  14.6  13.0  21.9   7.8  20.5   5.1  15.6
     2nd |  7.8  15.8  18.6  18.6   9.2  12.2   9.2   8.5
     3rd | 14.2  14.0  19.1  18.0   9.4  10.1   9.0   6.3
     4th | 20.1  11.6  15.6  14.4  10.1  10.4  11.4   6.4
     5th | 21.6  12.2  13.4  10.9  12.6   9.4  13.7   6.3
     6th | 19.1  12.1  11.3   8.3  15.3  10.8  15.7   7.5
     7th | 11.7  10.7   6.5   5.0  17.9  11.8  23.8  12.8
   Worst |  4.0   9.0   2.6   3.0  17.8  14.8  12.0  36.7
---------------------------------------------------------
```

mvmeta command is stored in F9

.
. sucra prob*, lab(A B C D E F G H)

Treatment Relative Ranking of Model 1

```
  +--------------------------------------+
  | Treatm~t | SUCRA | PrBest | MeanRank |
  |----------+-------+--------+----------|
  |        A |  46.3 |    1.6 |      4.8 |
  |        B |  54.9 |   14.6 |      4.2 |
  |        C |  61.3 |   13.0 |      3.7 |
  |        D |  66.6 |   21.9 |      3.3 |
  |        E |  40.4 |    7.8 |      5.2 |
  |        F |  52.9 |   20.5 |      4.3 |
```

```
 |        G |  39.8 |    5.1 |      5.2 |
 |        H |  37.7 |   15.6 |      5.4 |
  +-------------------------------------+
```

. graph save "Graph" "E:\Meta 分析\重要数据\重新分析\重新分析的 RCT\死亡\3.gph"
file E:\Meta 分析\重要数据\重新分析\重新分析的 RCT\死亡\3.gph saved

. netleague, lab(A B C D E F G H) sort(D C B F A
> E G H) export ("D:\cDEATH.xlsx") eform

Warning: The existing dataset is stored as a tem
> porary file
Warning: To save any changes applied at this tem
> porary file in a specific directory you need to
> use the 'Save as' menu

The league table has been stored at the end of t
> he dataset

.
. network convert pairs
Converting augmented to pairs ...

.
. netfunnel _y _stderr _t1 _t2 , random bycomp ad
> d(lfit _stderr _ES_CEN) noalpha

Comparisons in the plot:

1. G vs H
2. E vs F
3. C vs G
4. C vs D
5. B vs F
6. B vs C
7. A vs G
8. A vs F
9. A vs E
10. A vs D
11. A vs C

. graph save "Graph" "E:\Meta 分析\重要数据\重新分

> 析\重新分析\重新分析的 RCT\死亡\4.gph"
file E:\Meta 分析\重要数据\重新分析\重新分析的 RCT\
> 死亡\4.gph saved

.
