# Supplementary material for: Comparative Safety of Anticoagulant, Antiplatelet and the Combination of Both for Acute Coronary Syndrome: A Systematic Review and Network Meta-Analysis
Source: Biomedicines. 2025 Aug 20;13(8):2027. doi: 10.3390/biomedicines13082027 (PMC12383640; doi:10.3390/biomedicines13082027)
Supplement: Supplementary file 1 [file biomedicines-13-02027-s001.zip › raw data/Software operation records of emboilsm.pdf]

```
  ___  ____  ____  ____  ____ ©
 /__    /   ____/   /   ____/      17.0
___/   /   /___/   /   /___/       MP—Parallel Edition

 Statistics and Data Science       Copyright 1985-2021 StataCorp LLC
                                   StataCorp
                                   4905 Lakeway Drive
                                   College Station, Texas 77845 USA
                                   800-STATA-PC        https://www.stata.com
                                   979-696-4600        stata@stata.com

Stata license: Single-user 8-core , expiring  1 Jan 2025
Serial number: 501709301094
  Licensed to: 1
               1

Notes:
      1. Unicode is supported; see help unicode_advice.
      2. More than 2 billion observations are allowed; see help obs_advice.
      3. Maximum number of variables is set to 5,000; see help set_maxvar.

Running c:\ado\plus\profile.do ...

. *(4 variables, 38 observations pasted into data editor)

. network setup r n, studyvar(id) trtvar(t) format(augment) or
Treatments used
   A (reference):                   1
   B:                               2
   C:                               3
   D:                               4
   E:                               5
   F:                               6
   G:                               7
   H:                               8

Measure                             Log odds ratio

Studies
   ID variable:                     id
   Number used:                     19
   IDs with zero cells:             7 11 13 14 16
   - count added to all their cells:   .5
```

IDs with augmented reference arm: 2 4 6 7 8 12 13 14 17 18 19
- observations added: 0.00001
- mean in augmented observations: study-specific mean

Network information
Components: 1 (connected)
D.f. for inconsistency: 5
D.f. for heterogeneity: 7

Current data
Data format: augmented
Design variable: _design
Estimate variables: _y*
Variance variables: _S*
Command to list the data: list id _y* _S*, noo sepby(_design)

.
. network map
Graph command stored in F9

. graph save "Graph" "E:\Meta 分析\重要数据\重新分析\重新加的文章\栓塞\1.gph"
file E:\Meta 分析\重要数据\重新分析\重新加的文章\栓塞\1.gph saved

. set matsize 10000
set matsize ignored.
Matrix sizes are no longer limited by c(matsize) in modern Statas. Matrix sizes are now limited by edition of Stata. See limits for more
details.

.
. network meta i
Command is: mvmeta _y _S , bscovariance(exch 0.5) longparm suppress(uv mm) eq(_y_C: des_BC, _y_D: des_CD, _y_F: des_BF des_EF, _y_G: des_CG) vars(_
> y_B _y_C _y_D _y_E _y_F _y_G _y_H)
Note: using method reml
Note: regressing _y_B on (nothing)
Note: regressing _y_C on des_BC
Note: regressing _y_D on des_CD
Note: regressing _y_E on (nothing)
Note: regressing _y_F on des_BF des_EF
Note: regressing _y_G on des_CG
Note: regressing _y_H on (nothing)
Note: 19 observations on 7 variables
Note: variance-covariance matrix is proportional to .5*I(7)+.5*J(7,7,1)

```
initial:       log likelihood = -111.49643
rescale:       log likelihood = -110.51903
rescale eq:    log likelihood =  -108.9777
Iteration 0:   log likelihood =  -108.9777
Iteration 1:   log likelihood = -108.11477  (not concave)
Iteration 2:   log likelihood = -108.11073
Iteration 3:   log likelihood = -108.11069
Iteration 4:   log likelihood = -108.11069

Multivariate meta-analysis
Variance-covariance matrix = proportional .5*I(7)+.5*J(7,7,1)
Method = reml                              Number of dimensions    =     7
Restricted log likelihood = -108.11069         Number of observations  =    19
------------------------------------------------------------------------------
             | Coefficient  Std. err.      z    P>|z|     [95% conf. interval]
-------------+----------------------------------------------------------------
_y_B         |
       _cons |  -.7443723    .507743    -1.47   0.143     -1.73953    .2507858
-------------+----------------------------------------------------------------
_y_C         |
      des_BC |  -.3070728   .6382497    -0.48   0.630    -1.558019    .9438736
       _cons |  -.1560944   .3413307    -0.46   0.647    -.8250902    .5129014
-------------+----------------------------------------------------------------
_y_D         |
      des_CD |  -.5758279   .7317053    -0.79   0.431    -2.009944    .8582881
       _cons |   .4197335   .1330641     3.15   0.002     .1589327    .6805344
-------------+----------------------------------------------------------------
_y_E         |
       _cons |   .2521874   .3575711     0.71   0.481    -.4486391    .9530139
-------------+----------------------------------------------------------------
_y_F         |
      des_BF |  -.4127667   .8761399    -0.47   0.638    -2.129969    1.304436
      des_EF |   1.698946   .8306446     2.05   0.041     .0709129     3.32698
       _cons |  -.6358289   .4401473    -1.44   0.149    -1.498502    .2268439
-------------+----------------------------------------------------------------
_y_G         |
      des_CG |  -1.987278   1.600832    -1.24   0.214     -5.12485    1.150294
       _cons |   1.621048   1.551065     1.05   0.296    -1.418984     4.66108
-------------+----------------------------------------------------------------
_y_H         |
       _cons |   1.362326   1.700115     0.80   0.423    -1.969837     4.69449
------------------------------------------------------------------------------
```

```
Estimated between-studies SDs and correlation matrix
---------------------------------------------------
            SD       _y_B       _y_C       _y_D       _y_E       _y_F       _y_G       _y_H
_y_B  5.618e-07          1          .          .          .          .          .          .
_y_C  5.618e-07         .5          1          .          .          .          .          .
_y_D  5.618e-07         .5         .5          1          .          .          .          .
_y_E  5.618e-07         .5         .5         .5          1          .          .          .
_y_F  5.618e-07         .5         .5         .5         .5          1          .          .
_y_G  5.618e-07         .5         .5         .5         .5         .5          1          .
_y_H  5.618e-07         .5         .5         .5         .5         .5         .5          1
---------------------------------------------------

Testing for inconsistency:
 ( 1)  [_y_C]des_BC = 0
 ( 2)  [_y_F]des_BF = 0
 ( 3)  [_y_D]des_CD = 0
 ( 4)  [_y_G]des_CG = 0
 ( 5)  [_y_F]des_EF = 0

           chi2(  5) =    7.39
         Prob > chi2 =    0.1932
mvmeta command stored as F9; test command stored as F8


.
. network meta c
Command is: mvmeta _y _S  , bscovariance(exch 0.5) longparm suppress(uv mm)  vars(_y_B
_y_C _y_D _y_E _y_F _y_G _y_H)
Note: using method reml
Note: using variables _y_B _y_C _y_D _y_E _y_F _y_G _y_H
Note: 19 observations on 7 variables
Note: variance-covariance matrix is proportional to .5*I(7)+.5*J(7,7,1)

initial:       log likelihood = -120.01314
rescale:       log likelihood = -117.57619
rescale eq:    log likelihood = -115.97497
Iteration 0:   log likelihood = -115.97497
Iteration 1:   log likelihood = -115.57302
Iteration 2:   log likelihood = -115.57221
Iteration 3:   log likelihood =  -115.5722

Multivariate meta-analysis
Variance-covariance matrix = proportional .5*I(7)+.5*J(7,7,1)
Method = reml                               Number of dimensions    =     7
Restricted log likelihood = -115.5722          Number of observations  =    19
```

```
------------------------------------------------------------------------------
             | Coefficient  Std. err.      z    P>|z|     [95% conf. interval]
-------------+----------------------------------------------------------------
_y_B         |
       _cons |  -.3203353   .2640389    -1.21   0.225     -.837842    .1971714
-------------+----------------------------------------------------------------
_y_C         |
       _cons |  -.0185674   .2466452    -0.08   0.940    -.5019831    .4648483
-------------+----------------------------------------------------------------
_y_D         |
       _cons |   .4012068   .1306382     3.07   0.002     .1451607    .6572529
-------------+----------------------------------------------------------------
_y_E         |
       _cons |  -.0993554   .3190102    -0.31   0.755    -.7246038    .5258931
-------------+----------------------------------------------------------------
_y_F         |
       _cons |  -.3012961   .3212963    -0.94   0.348    -.9310253     .328433
-------------+----------------------------------------------------------------
_y_G         |
       _cons |  -.1981902   .3138932    -0.63   0.528    -.8134095    .4170291
-------------+----------------------------------------------------------------
_y_H         |
       _cons |  -.4569115   .7636202    -0.60   0.550     -1.95358    1.039757
------------------------------------------------------------------------------

Estimated between-studies SDs and correlation matrix
--------------------------------------------------
             SD        _y_B       _y_C       _y_D       _y_E       _y_F       _y_G       _y_H
_y_B  4.449e-07          1          .          .          .          .          .          .
_y_C  4.449e-07         .5          1          .          .          .          .          .
_y_D  4.449e-07         .5         .5          1          .          .          .          .
_y_E  4.449e-07         .5         .5         .5          1          .          .          .
_y_F  4.449e-07         .5         .5         .5         .5          1          .          .
_y_G  4.449e-07         .5         .5         .5         .5         .5          1          .
_y_H  4.449e-07         .5         .5         .5         .5         .5         .5          1
--------------------------------------------------
mvmeta command stored as F9

.
. network forest

. graph save "Graph" "E:\Meta 分析\重要数据\重新分析\重新加的文章\栓塞\2.gph"
file E:\Meta 分析\重要数据\重新分析\重新加的文章\栓塞\2.gph saved
```

```
. network rank max, all zero reps(5000) gen(prob)
Command is: mvmeta, noest pbest(max  in 1, zero id(id) all reps(5000) gen(prob) stripprefix(_y_) zeroname(A) rename(A = 1, B = 2, C = 3, D = 4, E =
> 5, F = 6, G = 7, H = 8))

Estimated probabilities (%) of each treatment having each rank
- assuming the maximum parameter is the best
- using 5000 draws
- allowing for parameter uncertainty
----------------------------------------------------------
          |                   Treatment
     Rank |    1     2     3     4     5     6     7     8
----------+-----------------------------------------------
     Best |  0.0   0.1   3.3  76.3   5.3   1.3   1.8  12.0
      2nd | 22.9   0.9  20.6  17.8  17.5   5.9   5.7   8.7
      3rd | 29.3   2.9  24.6   4.0  14.6   8.1  11.5   5.1
      4th | 21.7   7.3  25.0   1.3  14.1  10.5  15.3   4.8
      5th | 14.3  15.7  18.0   0.5  13.0  12.9  20.1   5.4
      6th |  7.9  27.0   7.1   0.1  13.5  16.0  21.4   7.1
      7th |  3.2  29.6   1.3   0.0  13.9  24.3  18.3   9.5
    Worst |  0.7  16.5   0.1   0.0   8.1  21.0   5.9  47.6
----------------------------------------------------------
mvmeta command is stored in F9

.
. sucra prob*, lab(A B C D E F G H)

Treatment Relative Ranking of Model 1

  +-----------------------------------------+
  | Treatm~t | SUCRA | PrBest | MeanRank |
  |----------+-------+--------+----------|
  |        A |  61.8 |    0.0 |      3.7 |
  |        B |  25.8 |    0.1 |      6.2 |
  |        C |  62.7 |    3.3 |      3.6 |
  |        D |  95.4 |   76.3 |      1.3 |
  |        E |  50.2 |    5.3 |      4.5 |
  |        F |  31.7 |    1.3 |      5.8 |
  |        G |  41.0 |    1.8 |      5.1 |
  |        H |  31.4 |   12.0 |      5.8 |
  +-----------------------------------------+

. graph save "Graph" "E:\Meta 分析\重要数据\重新分析\重新加的文章\栓塞\3.gph"
file E:\Meta 分析\重要数据\重新分析\重新加的文章\栓塞\3.gph saved
```

. netleague, lab(A B C D E F G H) sort(D C A E G F H B) export ("D:\cDEATH.xlsx") eform

Warning: The existing dataset is stored as a temporary file
Warning: To save any changes applied at this temporary file in a specific directory you need to use the 'Save as' menu

The league table has been stored at the end of the dataset

.
. network convert pairs
Converting augmented to pairs ...

.
. netfunnel _y _stderr _t1 _t2 , random bycomp add(lfit _stderr _ES_CEN) noalpha

Comparisons in the plot:

1. G vs H
2. E vs F
3. C vs G
4. C vs D
5. B vs F
6. B vs C
7. A vs G
8. A vs F
9. A vs E
10. A vs D
11. A vs C
12. A vs B

. graph save "Graph" "E:\Meta 分析\重要数据\重新分析\重新加的文章\栓塞\4.gph"
file E:\Meta 分析\重要数据\重新分析\重新加的文章\栓塞\4.gph saved
